# Treatment and Prognoses in Patients With Primary Gastrointestinal Stromal Tumors ≥10 cm: A Single-Institution Experience in China

**DOI:** 10.1097/MD.0000000000001117

**Published:** 2015-07-17

**Authors:** Chaoyong Shen, Haining Chen, Yuan Yin, Jiaju Chen, Sumin Tang, Bo Zhang, Luyin Han, Zhixin Chen, Jiaping Chen

**Affiliations:** From the Department of Gastrointestinal Surgery (CS, HC, YY, JC, ST, BZ, ZC, JC); and Intensive Care Unit, West China Hospital, Sichuan University, Chengdu, China (LH).

## Abstract

Data on treatments and specific outcomes of primary gastrointestinal stromal tumors (GISTs) ≥10 cm are limited. We here report the treatments and survival outcomes concerning a subgroup of primary giant GISTs.

Data of 83 consecutive patients with primary GISTs ≥10 cm in a single institution were retrospectively collected. Fifty-eight patients underwent surgery before imatinib mesylate (IM) treatment (Group A), 10 underwent surgical resection following IM therapy (Group B), whereas 15 patients took IM as drug therapy alone (Group C).

The baseline clinical characteristics were similar among the 3 groups. However, a lower proportion in Group A had metastatic disease at the time of diagnosis or surgery compared with Groups B and C (8.6% vs 40.0% vs 40.0%, *P* < 0.05). The median follow-up duration was 21.5 months. No statistically significant differences were observed on progression-free survival (PFS) among the groups. However, patients in Group B showed significantly better overall survival (OS) compared with those in Group C (*P* = 0.044). Multivariate analysis showed that patients treated with adjuvant IM were associated with better PFS (hazard ratio [HR] 3.01; 95% confidence interval [CI] 1.13–7.97; *P* = 0.027) and OS (HR 29.11; 95% CI 3.32–125.36; *P* = 0.004). The subgroup with mitotic count >10/50 high-power fields (HPF) showed worse PFS (HR 3.50; 95% CI 1.19–10.25; *P* = 0.022) and OS (HR 20.04; 95% CI 1.67–143.79; *P* = 0.018) than that of mitotic count ≤5/50 HPF.

Clinical treatment patterns for primary giant GISTs are different, and the outcomes of different interventions vary. The optimal treatments for these subgroup of patients still require further long-term investigation. Moreover, mitotic count and adjuvant IM are closely associated with PFS and OS in giant GISTs.

## INTRODUCTION

Gastrointestinal stromal tumors (GISTs) are a group of mesenchymal tumors found within the abdominal cavity (stomach, small intestine, and occasionally, mesentery, liver, retroperitoneum, and omentum), with an estimated incidence of 10 to 20 per million.^1–3^ Nowadays, surgery is the mainstay treatment for GIST patients. However, approximately 30% to 50% of GIST patients who underwent complete resection may experience tumor progression within 2 years postoperatively, particularly those with GISTs >10 cm,^4,5^ thereby affecting long-term outcomes. Combined resection may be adopted for GISTs ≥10 cm because of invasion into adjacent organs,^6,7^ and the proportion of tumor rupture and extensive intraoperative hemorrhage may also increase at surgery. As a consequence, treating giant GISTs is often a challenge for the attending clinicians.

The majority of GISTs harbor a gain-of-function mutation in either *KIT* or platelet-derived growth factor receptor alpha (*PDGFRA*).^8^ The introduction of imatinib mesylate, which targets *KIT* or *PDGFRA*-activated GISTs, has revolutionized the management of this disease.^9^ However, secondary resistance eventually develops in most cases.^10^ As such, surgical resection of residual tumors after preoperative IM treatment in patients with advanced or recurrent/metastatic GISTs is beneficial to improve their survival outcomes.^11,12^ Thus, a combination of preoperative IM treatment and surgical intervention has gained wider acceptance. However, data on clinical care for giant GISTs are still rare to date.

Numerous patients with GISTs ≥10 cm have been described, but most reports present case reports.^6,13–15^ In a retrospective study by Wada et al^7^, they reported the prognoses of this subset of patients, but no patient underwent neoadjuvant or adjuvant treatment until recurrence. Hence, clinical management and prognostic predictors remain unknown. To the best of our knowledge, studies that specifically target patients with primary GISTs ≥10 cm with such a large sample in a single institution are rare. Therefore, we aimed to explore the optimal treatment and evaluate the prognostic factors based on data of 83 patients with primary GISTs ≥10 cm who were consecutively admitted in our institution.

## MATERIALS AND METHODS

### Patient Selection

**FIGURE 1:**
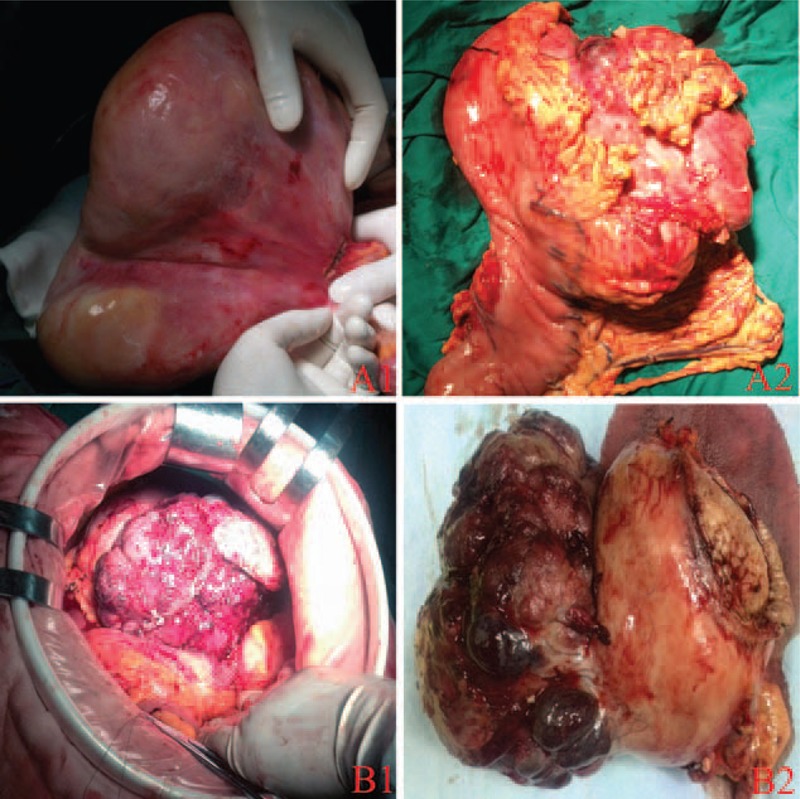
Gastrointestinal stromal tumors presented as a giant mass (15 cm × 15 cm) located in the greater curvature (A1, A2); total gastrectomy was performed for this patient. B1 and B2 showed a lesion located in the stomach with a size of 16 cm × 14 cm.

**FIGURE 2:**
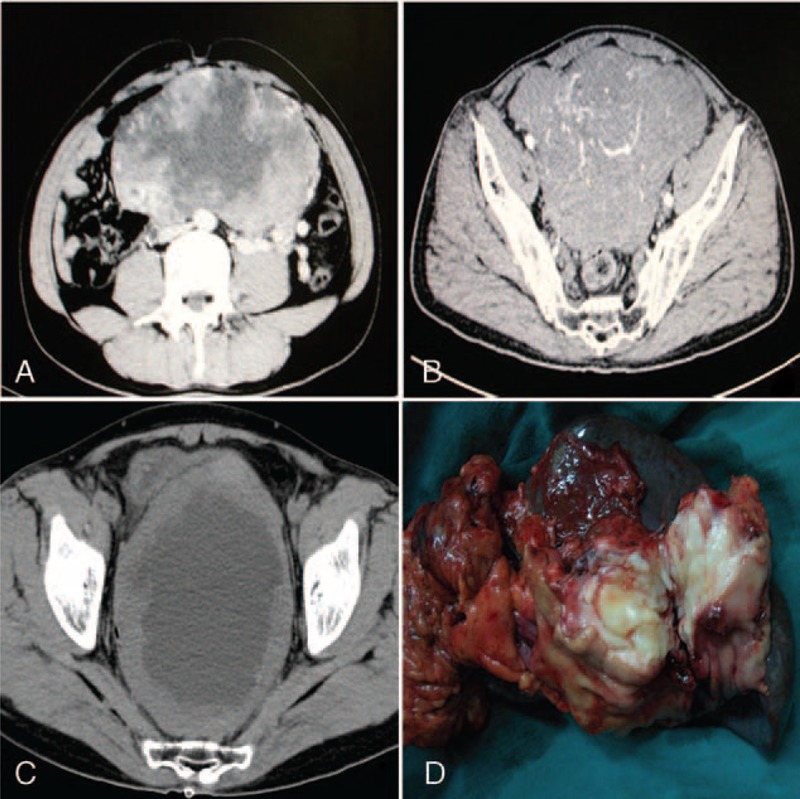
The computed tomography (CT) scan showed a giant tumor located in the abdominal cavity with a size of 22 cm × 16 cm (A). This patient underwent surgery after IM treatment, but eventually experienced tumor recurrence within 14 months postoperatively. Huge mass located in the pelvic cavity was observed by CT scans (B, C). The operative photo showed that the patient underwent proximal gastrectomy combined with splenectomy, which was attributed to the density adherent to the spleen (D).

The medical records of all consecutive patients with GISTs at the West China Hospital, Sichuan University between January 2011 and September 2014 were retrospectively reviewed. The inclusion criteria are as follows: patients were histologically diagnosed with primary GISTs by the pathologists at our institution; all tumors were ≥10 cm, as determined by computed tomography (CT) and intraoperative findings (Figures 1 and 2). Patients with GISTs synchronous with other malignancies and insufficient medical charts were excluded in this study. Data of 83 eligible patients with primary GISTs were collected during the specified period, from which 58 received surgical resection before IM therapy as initial treatment (Group A), 10 underwent surgery following IM treatment (Group B), whereas 15 took IM as drug therapy alone (Group C). Though surgery was recommended, the patients in Group C refused surgical resection because of their fear of potential surgical risks until last follow-up. Written informed consent was obtained from all patients. The institutional review board and Ethics Committee of the West China Hospital of Sichuan University deemed that an ethical review was not needed for this retrospective analysis.

### Surgery and IM Treatment

Patients with GISTs underwent surgical treatment with curative intent. However, combined resection should be performed for tumors that invaded adjacent tissues and organs in some patients (Figure 2). Frozen slices of incisal margin and surgical specimen were routinely collected during surgery. The surgery was classified into 3 categories: R0 (complete gross and microscopic resection), R1 (microscopic residual lesions), and R2 resections (the presence of any gross residual tumors). Patients who underwent preoperative IM therapy were confirmed as GISTs by endoscopic ultrasound-guided fine-needle aspiration biopsy or needle biopsies guided by CT, according to the National Comprehensive Cancer Network (NCCN) guidelines.^16^ The risk stratification of GISTs was evaluated according to the modified National Institutes of Health classification.^17^ The suggested IM dosage was 400 mg/day, and response was evaluated based on the Choi criteria.^18^

### Data Collection and Follow-Up

Data on age at diagnosis, sex, tumor location and size, preoperative and adjuvant IM duration, type of surgery, surgical outcome, mitotic count, and survival outcome of the patients were retrospectively collected. Follow-up was conducted by office visit, telephone call, or outpatient clinic visit from January 2015 to February 2015. Abdominal CT and ultrasonography, blood routine examination, and evaluation of liver and kidney functions were also performed.

### Survival and Statistical Analysis

Progression-free survival (PFS) was calculated as the number of months from the date of operation or administration of preoperative IM treatment to the day of disease progression. Overall survival (OS) was defined as the time from the start of treatment until death from any cause or last follow-up visit. All statistical analyses were performed using Statistical Package for Social Science (SPSS Inc, Chicago, IL). Measurement data were expressed as mean ± standard deviation. Differences among groups were analyzed using analysis of variance for continuous variables and *χ*^2^ test or Fisher exact test for categorical data. Survival analysis was performed using the Kaplan-Meier method and compared using a log-rank test, followed by a Cox proportional hazards regression model. Differences with 2-sided *P* < 0.05 indicated statistical significance.

## RESULTS

### Patient Characteristics

Data of 83 eligible patients with primary GISTs were collected, including 55 males (66.3%) and 28 females (33.7%), with a median age of 57 years (range, 26–87 years). Table 1 summarizes the baseline characteristics of the GIST patients. The tumor was located in the stomach, small intestine, and other parts (omentum, mesentery of small intestine and large intestine, retroperitoneal, and pelvic mass) in 33 (39.8%), 17 20.5%), and 33 (39.8%) cases, respectively. The mean tumor size for the entire cohort was 15.5 ± 6.1 cm; all tumors were classified as high risk. The proportion of patients with mitotic ≤5, 6 to 10, and >10/50 high-power fields (HPF) was 33.7% (n = 28), 36.1% (n = 30), and 30.1% (n = 25), respectively. The baseline clinical characteristics were similar among the 3 groups; however, a lower proportion in Group A had metastatic disease at the time of diagnosis or surgery compared with groups B and C (8.6% v 40.0% and 40.0%, *P* < 0.05). Among these patients, 8 had hepatic metastasis, whereas 7 had extensive abdominal cavity metastasis. The median time of preoperative medication among the 25 patients who received preoperative IM treatment was 8 months (range 1–29 months). All patients in Groups B and C achieved a partial response, whereas 46.7% (7/15) experienced tumor progression with a median medication time of 7 months (range, 2–29 months) in Group C. Adjuvant IM was administered to 27 patients (Group A, n = 20 v Group B, n = 7), with a median time of medication of 12 (range, 2–31) and 5 (range, 3–19) months, respectively.

**TABLE 1:**
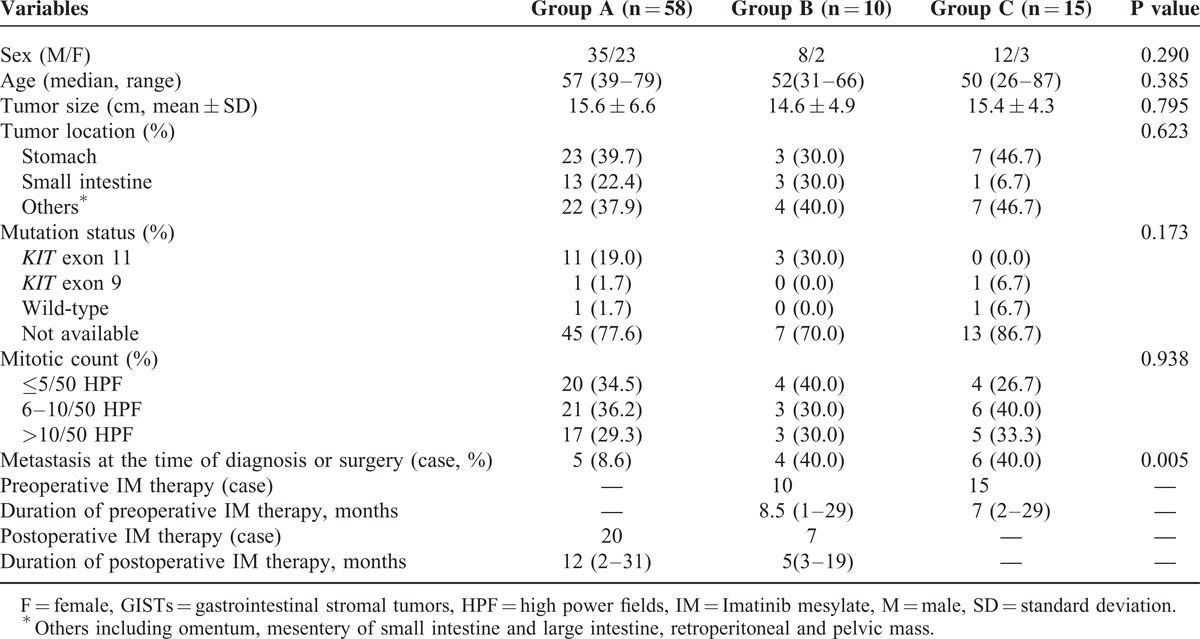
Clinicopathologic Characteristics of Patients With Primary Giant GISTs Among 3 Groups (n = 83)

### Surgical information

Table 2 describes the surgical interventions. A total of 68 patients underwent surgical resection (R0 resection, n = 62), in which partial/total gastrectomy was the most common procedure (n = 31, 45.6%). No significant differences were observed in tumor rupture at surgery, completeness of surgery, and type of surgery between Group A and B patients (*P* > 0.05). However, a trend for higher proportion of necessity for intraoperative transfusion and multivisceral resection was observed in Group A compared with Group B (20.7% vs 0.0% and 27.6% vs 10.0%, respectively); the differences were not significant (*P* > 0.05). The 30-day postoperative complications occurred in 5 patients, with an incidence of 7.4%, including wound dehiscence (n = 1), wound infection (n = 1), ileus (n = 2), and pulmonary infection (n = 1). One patient in Group B required reoperation because of wound dehiscence. No perioperative deaths were recorded.

**TABLE 2:**
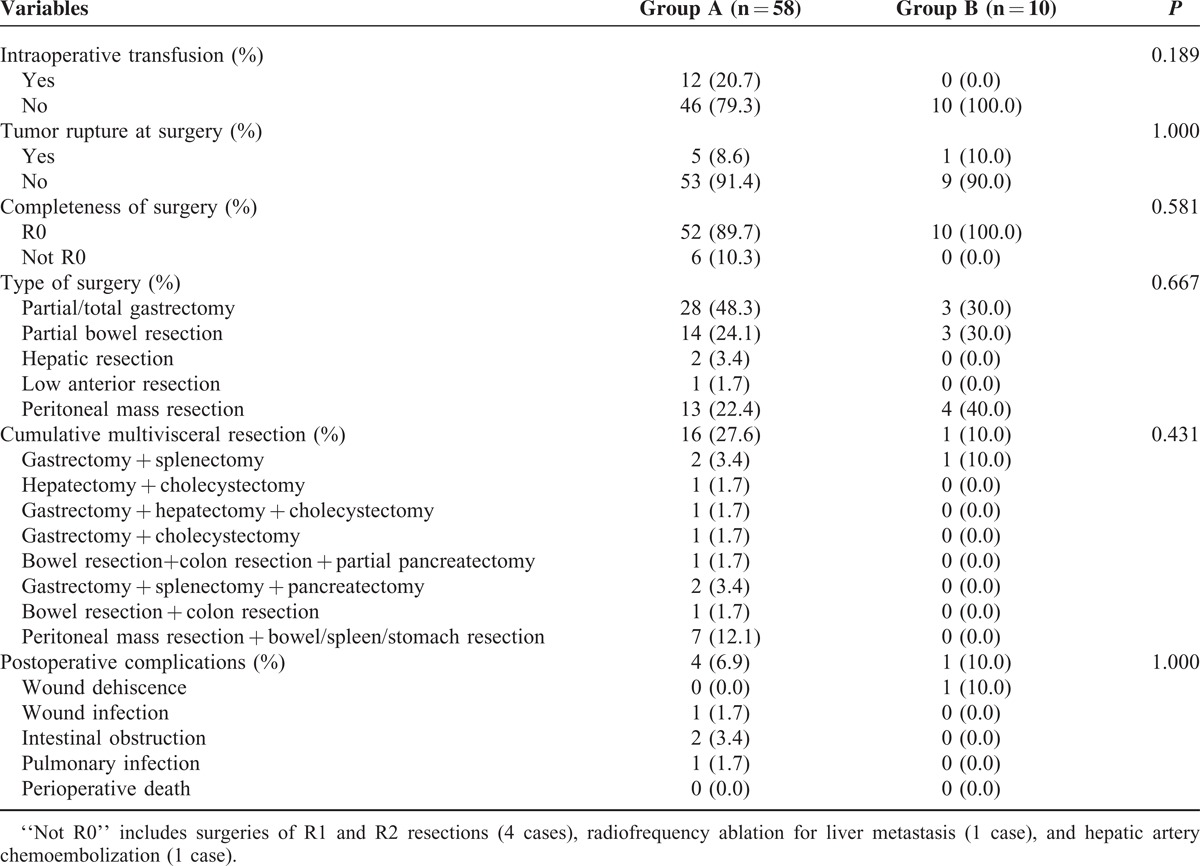
Surgery Information and Postoperative Complications Between 2 Groups (n = 68)

### Survival Outcomes

**FIGURE 3:**
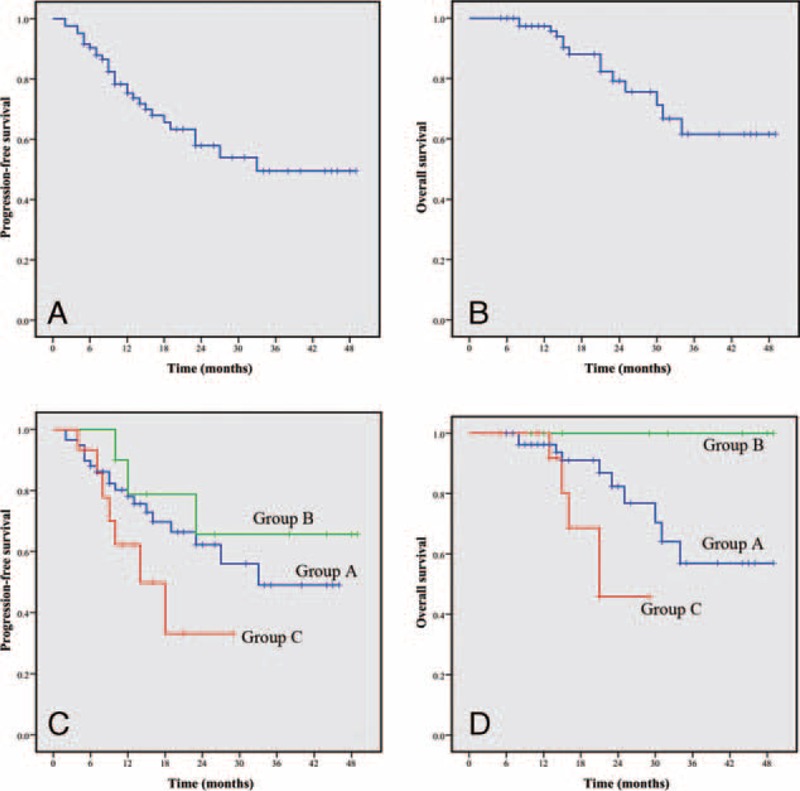
Progression-free survival (A) and overall survival (B) of patients with giant GISTs calculated by the Kaplan–Meier curve (n = 83). Progression-free survival (C) and overall survival (D) in all patients with tumors ≥10 cm were stratified by different treatments. The blue line refers to Group A, green line refers to Group B, and red line refers to Group C.

With a median follow-up duration of 21.5 months (range, 5–49 months), 29 patients experienced tumor progression (median time to progression was 10 months), whereas14 patients died for the entire cohort. The PFS rate for the entire cohort was 75.2% and 49.5%, whereas the OS rate was 97.4% and 61.6%, at 1 and 3 years, respectively (Figure 3A,B). The subgroups of patients who received different treatment strategies did not show significant differences on PFS; however, Group B patients revealed a trend of higher PFS rate than Groups C (*P* = 0.096) and A (*P* = 0.467) (Figure 3C). Moreover, Group B patients showed significantly better OS compared with Group C (*P* = 0.044), and revealed no statistical significance compared with Group A (*P* = 0.056) (Figure 3D). The details of the univariate and multivariate analyses by Cox regression model are listed in Table 3. Patients treated with adjuvant IM demonstrated significantly better PFS (hazard ratio [HR] 3.01; 95% confidence interval [CI] 1.13–7.97; *P* = 0.027) and OS (HR 29.11; 95% CI 3.32–125.36; *P* = 0.004). The mitotic count >10/50 HPF showed worse PFS and OS than the mitotic count ≤5/50 HPF ([HR 3.50; 95% CI 1.19–10.25; *P* = 0.022] and [HR 20.04; 95% C I 1.67–143.79; *P* = 0.018]). However, those of sex, age, tumor size and location, and preoperative IM therapy exhibited no notable differences on the prognosis of GISTs.

**TABLE 3:**
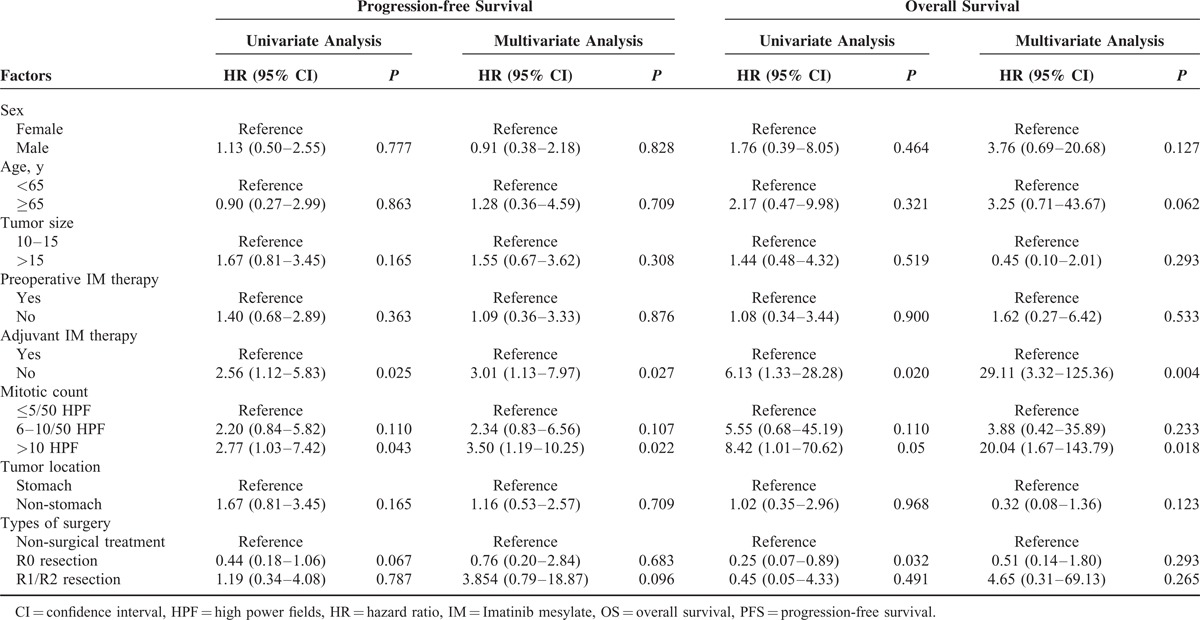
Univariate and Multivariate Analysis of Factors With PFS and OS Using Cox Proportional Hazards Regression Modeling

## DISCUSSION

The incidence of GISTs is increasing in the oriental population. However, available data specifically targeting patients with primary GISTs ≥10 cm are poorly described in clinical trials. This study describes, for the first time, the treatment and outcomes of giant GISTs with a large sample in a single institution. Our results suggest that patients who underwent preoperative IM treatment followed by surgical resection exhibit better OS compared with those who underwent IM therapy alone; meanwhile, a trend of better OS than the patients who underwent surgery before IM treatment was also observed. Moreover, mitotic count and adjuvant IM have been demonstrated to be closely associated with PFS and OS in giant GISTs (≥10 cm).

Resection of giant GISTs could be difficult, and sometimes considerable morbidity may occur at the time of surgical interventions. Giant GISTs are often supplied by multisource arteries and densely adherent to adjacent organs, which may cause failure of surgical resection. The proportion of concomitant adjacent organ invasion is as high as 26% with large gastric GISTs (≥10 cm), as previously reported in literature.^19^ In this study, approximately 25.0% (17/68) of the patients underwent multiorgan resection because of tumor invasion, which was in accordance with literature report. Primary liver GISTs are rare,^3^ but liver metastasis or involvement is common.^12^ Our data show that giant gastric GISTs are frequent in distant metastases; 15 patients had metastasis at the time of initial diagnosis or surgery, including 8 with hepatic metastasis and 7 with extensive abdominal cavity metastasis. Increasing evidence suggests that metastasectomy of liver increases the long-term OS and disease-free survival.^20^ The use of radiofrequency ablation and/or hepatic artery embolization as an adjunct to hepatectomy for metastasis in GISTs may achieve radical effects.^21,22^

Currently, surgery should be the initial treatment if the GISTs are technically resectable, which has an acceptable morbidity risk after preoperatively assessed by a multidisciplinary team. Otherwise, IM treatment before surgical resection for some patients with advanced/metastatic/recurrent/giant GISTs should be considered. The approval of IM has resulted in significant improvements in the survival outcomes for patients with GISTs.^9^ At most 80% of patients exhibit an initial response to IM; however, secondary resistance eventually develops in most cases.^10^ In this study, all patients (15 patients) who underwent IM treatment alone until last follow-up achieved a partial response, whereas 46.7% (7/15) experienced tumor progression, with a median medication time of 7 months (range, 2–29 months) in Group C. Data from previous publications show that removal of residual tumors after IM treatment could benefit the patients.^12,23^ In such situation, surgery creates the only opportunity to remove clones that may become resistant to IM. Consistent with the results of previous studies, the patients with primary giant GISTs who underwent surgery after IM treatment in this study showed a trend of better OS (*P* = 0.044) than those who underwent IM alone. Therefore, IM should be continued as a component of the best supportive care to limit the growth of sensitive clones in patients with progressive disease on IM, according to the NCCN task force recommendation.^24^ Careful exploration in operation is important to limit tumor rupture, specifically in giant masses with necrosis or cystic degeneration. Preoperative IM treatment could reduce the proportion of multiorgan resection, intraoperative bleeding, and downstage giant tumors and increase the opportunity of R0 resection.^25,26^ However, patients who received surgery after IM treatment in this study showed a trend of lower proportion of necessity of intraoperative transfusion and multivisceral resection than those who underwent surgical resection before IM therapy, although the differences were not significant (*P* > 0.05). This finding could be attributed to the small number of patients in Group B. Therefore, further study of surgical outcomes on giant GISTs treated with IM is needed.

Limited data concerning prognoses of patients with primary giant GISTs are available. In a retrospective study by DeMatteo et al, the 5-year recurrence-free survival (RFS) rate in 39 patients with giant GISTs based on subgroup analyses was approximately 45%,^27^ which was in accordance with the results obtained by Goh et al.^19^ In the present study, the PFS rate for the entire cohort was 49.5% at 3 years, whereas the OS rate was 61.6%. However, another study reported that patients with giant GISTs presented a 3-year RFS rate of 58.5%, and the 3-year OS rate was 88.1%,^7^ which was superior to our data. This phenomenon could be attributed to the fact that the research only consisted of patients who underwent resection, whereas patients who had peritoneal dissemination/distant metastasis or with tumor rupture at the time of surgery were excluded. Several factors have been reported as prognostic indicators, such as mitotic index, GISTs size, tumor location, gene mutation, and type of surgery,^7,12,27–29^ as well as body mass index.^30^ In the present cohort of primary giant GISTs, adjuvant IM and mitotic count were the 2 independent predictors of PFS and OS. Furthermore, tumor location is commonly considered as a prognostic factor; however, no significant differences in survival were observed between the tumor site of stomach and non-stomach, which is in agreement with previous studies.^7,12^ Patients older than 65 years presented a trend of worse OS than younger patients, although statistical significance was not reached (*P* = 0.062). Therefore, older age is associated with poor RFS in GIST patients,^31^ and may somehow affect long-term survival.

This study has some limitations. The findings should be carefully interpreted because of the retrospective nature and small sample of this study. Moreover, we were unable to explore the association between genetic mutation and survival because of limited available data. Hence, further randomized research based on large populations is required to provide more valuable information about the primary giant GISTs.

In conclusion, treatment patterns for primary giant GISTs are different, and the outcomes of different interventions vary. The optimal treatments for these subgroup patients still require further long-term investigation. Giant gastric GISTs are frequent in distant metastases. Moreover, we have also demonstrated that mitotic count and adjuvant IM are closely associated with PFS and OS in primary giant GISTs.
